# Canine and feline parasitic zoonoses in China

**DOI:** 10.1186/1756-3305-5-152

**Published:** 2012-07-28

**Authors:** Jia Chen, Min-Jun Xu, Dong-Hui Zhou, Hui-Qun Song, Chun-Ren Wang, Xing-Quan Zhu

**Affiliations:** 1 State Key Laboratory of Veterinary Etiological Biology, Key Laboratory of Veterinary Parasitology of Gansu Province, Lanzhou Veterinary Research Institute, Chinese Academy of Agricultural Sciences, Lanzhou, Gansu Province 730046, PR of China; 2 College of Veterinary Medicine, South China Agricultural University, Guangzhou Guangdong Province 510642, PR of China; 3 College of Animal Science and Veterinary Medicine, Heilongjiang Bayi Agricultural University, Daqing Heilongjiang Province 163319, PR of China; 4 College of Animal Science and Technology, Yunnan Agricultural University, Kunming Yunnan Province 650201, PR of China

**Keywords:** Parasitic zoonoses, China, Dogs, Cats, Prevalence

## Abstract

Canine and feline parasitic zoonoses have not been given high priority in China, although the role of companion animals as reservoirs for zoonotic parasitic diseases has been recognized worldwide. With an increasing number of dogs and cats under unregulated conditions in China, the canine and feline parasitic zoonoses are showing a trend towards being gradually uncontrolled. Currently, canine and feline parasitic zoonoses threaten human health, and cause death and serious diseases in China. This article comprehensively reviews the current status of major canine and feline parasitic zoonoses in mainland China, discusses the risks dogs and cats pose with regard to zoonotic transmission of canine and feline parasites, and proposes control strategies and measures.

## Review

### Background

Pet dogs and cats are often considered to be the faithful friends and intimate companions of humans, and enjoy life together with humans. This human-animal bond can provide substantial positive benefits with regard to emotional development, socialization and psychological and physiological well-being of humans [1]. However, dogs and cats also act as reservoirs of a large number of pathogens of parasitic zoonoses, such as toxoplasmosis [2], giardiasis [3], toxocariasis [4] and ancylostomiasis [5]. Their roles in transmitting human infections have been recognized worldwide [6,7].

With a major socioeconomic development and a significant increase in living standards, more and more dogs and cats are being raised and kept as family pets and companion animals by Chinese families, and the populations of dogs and cats are approximately 200 and 100 million in China, respectively [8]. In the country, some of the dogs and cats roam freely in urban environments or rural settings, so the presence of these animals in close contact with people constitutes a high potential risk. More importantly, the current status in the lack of zoonotic awareness and enough veterinary resources increases the risk of exposure to parasitic zoonoses, which poses a significant public problem in China.

Unfortunately, up to date there has been no published overview on canine and feline parasitic zoonoses in mainland China in the English literature, except for a few articles published in the Chinese language [9,10]. It is therefore timely to review comprehensively the current status of major canine and feline parasitic zoonoses in mainland China, discuss the risks dogs and cats pose with regard to zoonotic transmission of canine and feline parasites, and propose control strategies and measures.

### Zoonotic nematode infections

#### Toxocariasis

Human toxocariasis results from zoonotic transmission of the round worms, *Toxocara canis* (of dogs) and *T. cati* (of cats). Infection occurs when humans accidently ingest embryonated eggs through contaminated soil, food, fomites or by direct contact with dogs and cats [4], this can result in clinical syndromes such as visceral larva migrans (VLMs), ocular larva migrans (OLMs), eosinophilic meningoencephalitis (EME), covert toxocariasis (CT) and neurotoxocarosis [4,10-12].

Toxocariasis is one of the most common human parasitic infections in the world and high prevalence has been reported in developing countries, such as Indonesia and Brazil [13,14]. However, it is one of the most neglected parasitic infections in China. Only 20-years-ago literature was available on the seroprevalence of toxocariasis in humans in Chengdu, Sichuan Province, China [15]. A single retrospective diagnosis of patients ascertained that contact with infected dogs is the risk factor for human infections in China [16].

Widespread prevalence of *Toxocara* spp*.* in dogs and cats can lead to the contamination of surroundings with *Toxocara* eggs, which threatens public health directly [17,18]. Although only two comprehensive surveys of *Toxocara* infection in dogs were conducted in Hunan province [19] and Heilongjiang province [20] (Table 1), the results indicated that *T. canis* was actually the most common parasite species in dogs in Hunan province (45.2%) [19], and the second most common parasite in dogs in Heilongjiang province (36.5%) [20]. With regard to *Toxocara* of cats, in addition to *T. cati*, we have proven the existence of *T. malaysiensis* in cats in Guangzhou, China, and this parasite is remarkably different from *T. canisT. cati* and *T. leonina* of dogs and cats by molecular characterization [21]. However, a comprehensive epidemiological survey of *Toxocara* infections in cats in China has not yet been performed. Thus, the role of *T. cati* and *T. malaysiensis* as zoonotic parasites has yet to be assessed in future studies.

**Table 1:** The prevalence of major canine and feline parasitic zoonoses in mainland China

**Disease**	**Parasite**	**Prevalence in people**	**Prevalence in dogs and cats**	**Reference**
Toxocariasis	*Toxocara canis*, *T. cati*	2.1%–17.7% in children in Chengdu; a case reported in Yunnan province	45.2% in dogs in Hunan; 36.5% in dogs in Heilongjiang	[15,16,19,20]
Ancylostomiasis	*Necator americanus*, *Ancylostoma duodenale*, *Ancylostoma caninum*	6.12% in south China	66.3% in dogs in Heilongjiang; 20.3% in dogs in Hunan; Some reported cases in Gansu, Guangdong, Jiangsu and Inner Mongolia in dogs and cats	[9,19,20,24]
Strongyloidiasis	*Strongyloides stercoralis*	11.7% in small village of Yunnan province; some cases reported in Guangxi Zhuang Autonomous Region	Not available	[9,37]
Trichinellosis	*Trichinella spiralis*, *T. nativa*	3.19% in ten endemic provinces; 9 outbreaks by eating dog meat in north China	Canine trichinellosis reported in 11 endemic provinces; feline trichinellosis reported in 10 endemic provinces	[40,42,43]
Echinococcosis	*Echinococcu granulosus*, *E. multilocularis*	~0.4 million in 22 endemic provinces	39.9% in dogs in Qinghai province	[38,44]
Clonorchiasis	*Clonorchis sinensis*	2.4% in 27 endemic provinces; ~12.5 million; 31.90%–57.26% in some villages in Heilongjiang province; 15.2% in some villages of the Korean minority in Liaoning province	41.8% in dogs in Guangdong; 20.5% in cats in Guangdong	[24,38,48,51-54]
Paragonimiasis	*Paragonimus westermani*	1.71% in 24 endemic provinces	Dogs: 14.29% in Hubei; 9.9%–84.62% in Liaoning; 10% in Zhejiang; Cats: 4.5% in Guangxi; 56.25% in Hubei; 2.56%–5.6% in Jiangxi	[24,55]
Giardiasis	*Giardia lamblia*	2.5%; ~30 million	25.2% in dogs in Jilin Province; 13.1% in dogs Henan Province; 11% in pet dogs in Guangzhou	[38,77,78,80]
Toxoplasmosis	*Toxoplasma gondii*	7.9 –12.3% nationwide	21.3% in pet dogs in Guangzhou; 31.3% in stray dogs in Guangzhou; 30.77% in stray cats in Guangzhou; 17.98% in pet cats in Guangzhou; 10.81% in dogs in Lanzhou; 21.3% in cats in Lanzhou	[62,68-71]
Leishmaniasis	*Leishmania infantum Leishmania donovani*	More than 400 cases reported in endemic regions in western China	24.8% in dogs in Sichuan Province; 59.43% in dogs in Jiuzhaigou County; 37.21% in dogs in Beichuan County	[85-88,91-93]

#### Ancylostomiasis

Ancylostomiasis caused by blood-feeding hookworms is a neglected tropical disease in a number of countries with major socio-economic significance [22]. The common hookworms of dogs include *Ancylostoma caninumA. braziliense, A. ceylanicum* and *Uncinaria stenocephala*, while *A. tubaeformeA. brazilienseA. ceylanicum* and *U. stenocephala* are from cats [5]. *A. ceylanicum* was first isolated and identified in civet cats in Fujian province, China in 1911 [23] and human infection with *A. ceylanicum* was reported in Fujian in 1981, which documented that a high prevalence of *A. ceylanicum* in cats and dogs was the significant risk factor for human infection [23]. The parasites *Necator americanus* and *A. duodenale* are now found to be the most prevalent hookworms distributed in south China, such as Hainan, Guangxi, Fujian provinces and Chongqing Municipal, with a mean prevalence of 6.12% nationwide [24]. In particular, these two most common species are more common in Sichuan [25] and Yunnan [26,27] provinces. The infected people mainly live in less developed rural areas without inadequate sanitary conditions, where dogs and cats are known to roam freely and farmers often walk barefoot [28,29].

Hookworm infections in dogs and cats are endemic in Hunan, Gansu, Guangdong, Heilongjiang, Jiangsu provinces and Inner Mongolia Autonomous Region [9,19,20]. Although it has been documented that *A. caninum* (of dogs) and *A. tubaeforme* (of cats) are the most common species throughout the warmer ranges [30,31], *A. caninum* is also prevalent in the cold regions in China, such as Heilongjiang province, where it has the highest prevalence of 66.3% [20].

#### Strongyloidiasis

Strongyloidiasis is caused by the nematode *Strongyloides stercoralis*, which is able to infect dogs, cats, monkeys and humans [32]. *S. stercoralis* is prevalent in endemic tropical and subtropical regions [33], especially in rural areas where sanitary conditions are inadequate and *S. stercoralis* filariform larvae contained in feces is able to cause human and dog infection [34,35]. Nonetheless, *S. stercoralis* is a neglected soil-transmitted helminth worldwide [36], with a paucity of epidemiologic data in China.

A few human infections have been reported mainly in tropical regions, such as Guangxi Zhuang Autonomous Region [9], where most cats and dogs usually roam freely in villages and cities. An 11.7% prevalence of *S. stercoralis* was found in a small village of Yunnan province where sanitary conditions were poor [37]. Therefore, it has been speculated that infected dogs and cats may represent a zoonotic risk and, thus, surroundings contaminated by feces of these animals may cause the infection of humans in the country. However, there is no available epidemiologic data on this disease of dogs and cats, and little is known of the dynamics of transmission of strongyloidiasis between dogs and cats and humans in China.

#### Trichinellosis

Human trichinellosis caused by *Trichinella spiralis* and *T. nativa* is one of the most widespread zoonosis in China [38]. Humans become infected through the consumption of raw or undercooked meat [39]. Between 2004 and 2009, seroepidemiological surveys of human infection with *Trichinella* indicated an average seroprevalence of 3.19%, with the highest seroprevalences mainly in western China, such as 8.43% in Yunnan province, 6.37% in Inner Mongolia Autonomous Region and 5.35% in Sichuan province [40].

In addition to infected pork being the main source of human infection, dog meat has become another important source of human infection with *Trichinella* in China [41]. Resulting from the consumption of dog meat, nine outbreaks of human trichinellosis occurred in north China, where the prevalence of *T. nativa* infection in dogs bred and farmed for human consumption was high [42]. Some *Trichinella* isolates from dogs and cats in northeastern China were identified as *T. nativa* by molecular approaches [43]. Epidemiological surveys have estimated that canine trichinellosis is distributed in 11 provinces/Autonomous Regions/Municipals/Special Administrative Regions (P/A/M/S) [43] (Table 1). *Trichinella* infection in cats has been recorded in 10 P/A/M [43] (Table 1).

### Zoonotic cestode infections

#### Echinococcosis

Echinococcosis is a worldwide parasitic zoonosis caused by infection with adult or larval stages of tapeworms of the genus *Echinococcus*. Among the seven recognized species of *Echinococcus*, both *E. granulosus* and *E. multilocularis* are significant for public health by causing cystic echinococcosis (CE) and alveolar echinococcosis (AE), respectively [44]. Both CE and AE are considered among the most serious parasitic zoonoses in China, and approximately 0.4 million people are infected nationwide [38].

CE and AE are prevalent in 22 provinces, and the main endemic areas include western and northwestern provinces and autonomous regions, such as Xinjiang, Gansu, Ningxia, Inner Mongolia, Qinghai, Tibet, and Sichuan [44] (Table 1). In northwestern China, dogs are the most important definitive host transmitting *E. granulosus* to humans [45] (Table 1), owing to the traditional practice of feeding dogs by herdsmen with offal (e.g., lungs and liver) of sheep and yaks during slaughtering seasons and their close relationship with local people. Moreover, it is documented that domestic dogs are also the predominant definitive host in the semidomestic cycle and are responsible for human AE in China [45-47].

Dogs are generally highly susceptible to *E. multilocularis* and a large number of owned and stray dogs in rural regions are usually poorly fed and live freely in the country. Therefore, dogs may get infected with the parasite from small mammals in the field, and AE is therefore easily transmitted to humans via eggs in the feces of dogs.

### Zoonotic trematode infections

#### Clonorchiasis

Clonorchiasis is a parasitic zoonosis caused by the oriental liver fluke *Clonorchis sinensis*. Humans and other mammals become infected with *C. sinensis* through ingestion of raw or undercooked freshwater fish and shrimp infected by *C. sinensis* metacercariae [48,49].

This infectious disease is regarded as one of the major parasitic zoonoses in some parts of Asia, including Korea, Japan, northern Vietnam, Thailand and China [48,50]. Human clonorchiasis has been reported in 27 provinces and autonomous regions in China [24], with approximately 12.5 million people being infected nationwide [38] (Table 1). Southern China’s Guangdong province has the largest number of infected populations (~5.5 million), which results from the local custom of consumption of raw and undercooked fish [48]. In the northeast, the prevalence of *C. sinensis* in some villages of Zhaoyuan County in Heilongjiang province ranged from 31.90% to 57.26% [51], and 15.2% of the Korean minority were infected with *C. sinensis* in some villages in Liaoning province [52].

In addition to humans, cats and dogs serve as definitive hosts for *C. sinensis*, and they are considered to be the most important reservoir hosts for human infection in the endemic regions of China [53,54] (Table 1).

#### Paragonimiasis

*Paragonimus westermani*, the lung fluke, is of major socioeconomic importance in Asia [55]. Humans become infected by consuming raw or undercooked stream crabs that are infected by *P. westermani*, drinking water contaminated by metacercaria or undercooked meat from a paratenic host [56]. The recent national survey indicated that paragonimiasis is endemic in 24 P/A/M, and the nationwide prevalence is estimated to be 1.71% [24] (Table 1). People in some P/A/M such as Hubei, Zhejiang and Fujian provinces have the higher prevalence [57-59].

Many carnivorous animals such as cats and dogs serve as definitive hosts of *P. westermani*, and cats and dogs are considered to be the most important reservoir hosts for human infections in endemic regions in China [60]. Some recent surveys have indicated that cat and dog infections are extremely severe in rural areas in Fujian and Hubei provinces [55] (Table 1) where most cats and dogs usually roam freely in villages and, thus, freely drink contaminated water or ingest crabs or crayfish infected with lung flukes [55,58].

### Zoonotic protozoan infections

#### Toxoplasmosis

Toxoplasmosis is an important zoonotic parasitic disease in humans and many species of birds and mammals, which is caused by the opportunistic protozoan *Toxoplasma gondii*[61]. It has been estimated that up to one third of the world’s population has been infected [61]. In China, the prevalence of human toxoplasmosis appears to be increasing, from 7.9% to 12.3% between 2001 and 2008 [38,62,63] (Table 1).

As the definitive hosts for *T. gondii*, cats can pass oocysts in their feces leading to contamination of *T. gondii* oocysts in soil [2,64]. In China, owing to excretion of *T. gondii* oocysts in the environment by stray cats in parks, the possibility of human infection has increased [64]. As a risk factor for *T. gondii* infection in humans, the potential role of dogs has been recognized because of mechanical transmission of oocysts [61]. A recent study has also found that taking care of pet animals was a risk factor associated with *T. gondii* infections in humans [65]. People owning dogs and cats as pets also showed a high risk of infection, with the prevalence ranging from 5.3% to 34.8% in China [66,67]. Our recent serologic surveys using ELISA revealed that *T. gondii* infection in dogs in Guangzhou, southern China is high, especially in stray dogs, which have a prevalence of 21.3% and 31.3%, respectively [68]. Moreover, it was found that the prevalence of stray and household cats in Guangzhou China was 30.77% and 17.98%, respectively [69]. In Lanzhou, northwest China, a *T. gondii* prevalence of 10.81% in pet dogs [70] and 21.3% in pet cats was reported [71], indicating the widespread prevalence of *T. gondii* in China.

#### Giardiasis

Giardiasis caused by *Giardia* spp. is one of the most common human parasitic diseases that can cause public health problems in most developing countries as well as some developed countries [72,73]. In China, *G. lamblia* (syn. *G. duodenalis* and *G. intestinalis*) has been documented in every mainland province [38,74] (Table 1). However, there is still no detailed data on human infections nationwide, and thus *G. lamblia* infection is underestimated, despite it being documented that HIV positive individuals are susceptible to co-infections with *Giardia* (1.3% from 302 HIV positive individuals) in a rural village of Fuyang, Anhui province [75].

Most *Giardia* spp. are host adapted (narrow hosts range), but *G. lamblia* is considered to be a zoonotic agent that causes giardiasis in humans and most mammals including dogs and cats [74,76]. Until recently, in addition to some reports of prevalence of *Giardia* in dogs in China [77,78] (Table 1), there were only two recent studies on genotyping or subtyping of *Giardia* isolates from humans and animals [79,80]. By sequence analysis of the triosephosphate isomerase (tpi) gene of *Giardia duodenalis*, the Assemblages A and B were found in 12 and 6 human specimens, respectively in Henan province, China [79]. A very recent survey of *G. duodenalis* prevalence in pet dogs in Guangzhou, China revealed an 8.61% (18/209) prevalence using microscopy examination and 11% (23/209) using PCR [80]. The *G. duodenalis* prevalence was significantly higher in diarrheic dogs and young dogs than in non-diarrheic dogs and adult dogs. Assemblage D (18/23) and zoonotic Assemblage A (5/23) were found based on sequence analysis of the 23 PCR-positive samples, which indicated the potential risk of *G. duodenalis* transmission from pet dogs to humans in China, at least in Guangzhou [80].

#### Leishmaniasis

Leishmaniasis is one of the most important vector-borne diseases in the world caused by *Leishmania* spp. [81,82]. Humans and animals can become infected with *Leishmania* spp. through transmission of flagellated promastigotes by the insect vectors, phlebotomine sand flies [83]. In China, leishmaniasis caused by *Leishmania donovani* is still an important parasitic disease [84], and serological surveys reported that human cases occurred in western China, such as Sichuan, Shaanxi, Shanxi, Sichuan and Gansu provinces, and Inner Mongolia and Xinjiang and Autonomous Regions [85,86]. In particular, Xinjiang Autonomous Region is the most prevalent area in the country [87,88] (Table 1).

Canine leishmaniasis (CanL) is caused by *Leishmania infantum* (syn. *Leishmania chagasi*), which is mainly prevalent in regions of Europe, Africa, Asia and Latin America [89]. The domestic dog is the main reservoir for human infection [89,90]. In China, information regarding prevalence of CanL is very limited, and the only three available surveys were conducted in western China’s Sichuan province by PCR and serological tests [91-93] (Table 1). While it has confirmed that infected dogs are the major source of human infection with *Leishmania* spp. and some sporadic CanL reports have suggested that dog infections are mainly distributed in endemic western areas in the country [85,91], it is unclear whether dog infection contributed to the re-emergence of human leishmaniasis in western China due to lack of information on CanL prevalence in these endemic areas.

### Zoonotic ectoparasite infections

Sarcoptic mange caused by *Sarcoptes scabiei* is the most common ectoparasite infection in various animals including companion animals [94]. Also, scabies caused by *S. scabiei* in humans is one of the most common human health problems [94]. This global parasitic zoonosis is an emerging/re-emerging infectious disease seriously threatening human and animal health, causing significant public health concern [94]. Scabies is also prevalent in developing countries such as Brazil, where it has been shown to be a significant public health problem [95]. However, survey of scabies prevalence in China is scant, apart from several reports of canine and feline sarcoptic mange [96,97]. Obviously, scabies and sarcoptic mange are underestimated in China, dogs and cats pose potential health hazards by transmitting *S. scabiei* between themselves and humans.

#### Zoonotic risks

With an increasing number of dogs and cats, indirect or direct contact with animals is very common, therefore a large number of parasitic zoonosis can potentially be transmitted to humans from dogs and cats. In China, there are more than 1.3 billion people of 56 nationalities, each practices different hygiene habits, culture and customs. Some of the eating habits or customs are risk factors leading to the prevalence of parasitic zoonoses. For example, the prevalence of echinococcosis in vast western and northwestern pastoral regions in China is closely related to the unique practice of feeding dogs, while occurrence of trichinellosis in north China is partly owing to the custom of eating raw or undercooked meats of various kinds. In addition, pet ownership is an important risk factor for the occurrence of many parasitic zoonoses, such as toxocariasis [7], toxoplasmosis [2] and ectoparasite infections [94]. However, in China, most pet owners have not been educated about the presence of this risk factor, thus increasesing the possibility of exposure to parasitic zoonoses.

Since the faecal-oral route (via water or food) is a major transmission model for soil-transmitted helminth infections, widespread contamination of environment or drinking water with parasite eggs, oocysts, or infective larvae may induce the occurrence of parasitic zoonoses, in particular in rural areas where stray dogs and cats enjoy water sources together with humans.

#### Perspectives for control

As a result of uncontrolled populations of dogs and cats existing in close proximity to the increasing densities of human populations, effective control of canine and feline parasitic zoonoses is an extremely tough task in China. Given that a large number of canine and feline parasitic zoonoses have not been given high priority and the owners of dogs and cats also lack the related knowledge, the most common and important control strategies and approaches are to improve the public awareness of canine and feline parasitic zoonoses using various educational media, such as TV and radio, and to educate people to change their unhealthy eating habits, in particular for some ethnic groups. Furthermore, regulating the populations of dogs and cats, especially stray dogs and cats, promoting the significance of deworming dogs and cats, and improving the sanitation and hygiene of dogs and cats are among the recommended strategies and measures that can be taken to control canine and feline parasitic zoonoses in China**.**

## Competing interests

The authors declare that they have no competing interests.

## Authors' contributions

XQZ and JC conceived and designed the review, and critically revised the manuscript. JC drafted the manuscript. MJX, DHZ, HQS and CRW contributed to drafting the manuscript. All authors read and approved the final manuscript.
